# Development and characterisation of antimicrobial epoxy resin

**DOI:** 10.1038/s41598-025-90465-7

**Published:** 2025-04-23

**Authors:** Madeline Berrow, Alexander Brooks, Anna M. Kotowska, Julie Watts, Lily Riordan, Luke Kidger, David J. Scurr, Naa Dei Nikoi, Manuel Banzhaf, Jack Alfred Bryant, Simon Greenway, Violaine Mendez, Brian Norton, Felicity de Cogan

**Affiliations:** 1 School of Pharmacy, University of Nottingham, Nottingham, UK; 2 Institute of Microbiology and Infection, University of Birmingham, Birmingham, UK; 3 Indestructible Paint Ltd, 16-25 Pentos Drive, Birmingham, UK

**Keywords:** Chlorhexidine, C_7_H_4_N_2_Cl^−^, Antimicrobial, Polymer, Epoxy resin, Biocidal, Paint, Antimicrobials, Applied microbiology, Biomedical engineering

## Abstract

Surface contamination is an important, if under-discussed, route of infection transmission. In this study, we suspended chlorhexidine digluconate (CHX) in epoxy resin. CHX was found to be stably incorporated into the material, and its addition to epoxy resin was found to have minimal effects on the optical transparency of the material. After application of the epoxy resin to steel surfaces, time-of-flight secondary ion mass spectrometry revealed that CHX was uniformly present over the surface. Surfaces painted with CHX-resin were found to have significant, reproducible antimicrobial efficacy against *E. coli*, *S. aureus*, and *C. albicans*. We have shown that the addition of CHX has minimal effects on the adhesion of the epoxy resin to surfaces, as well as a high durability of the antimicrobial efficacy. We believe that this material has a wide array of applications, and could be utilised to confer significant, low-cost antimicrobial efficacy to existing surfaces, to prevent surface contamination, and to stop the transmission of infectious disease.

## Introduction

Contamination of surfaces by pathogenic microorganisms is an important route of infection with this being especially important in healthcare settings. Research has suggested that healthcare professionals frequently encounter pathogenic microorganisms through contact with contaminated surfaces—one study suggested that 52% of healthcare personnel acquired vancomycin-resistant *Enterococci* through contact with surfaces^1^, whilst another study claimed that 40% of healthcare personnel who touched contaminated surfaces acquired methicillin-resistant *Staphylococcus aureus* on their hands^2,3^. Recent studies have suggested that SARS-CoV-2 can persist on stainless steel for 48 h and can remain viable on plastic for up to 72 h^4,5^. This is also observed with bacterial species, as *Escherichia coli* can survive for more than 28 days on stainless steel^6^, and *S. aureus* has been found to persist on stainless steel for six weeks^7^, and can persist on polyethylene for over 90 days^8^. The pathogenic bacteria *Acinetobacter baumannii*, which can cause nosocomial infections, has been found to be able to survive for seven days on glass, and for more than 25 days on cotton^9^, although other studies have found that strains associated with hospital outbreaks can survive for up to 33 days on glass surfaces^10^. *Klebsiella pneumoniae*, another frequent cause of nosocomial infections, can survive for seven days on stainless steel and polycarbonate surfaces, and can remain viable on aluminium for more than 15 days^11^. The pathogenic fungus *Candida albicans* has been found to survive for three days on both glass and steel surfaces, and could survive on fabric surfaces for 14 weeks^12^, whilst *Aspergillus fumigatus* has been found to remain viable on plastic surfaces for up to a month^13^.

Despite the clear prevalence of pathogenic microorganisms in hospital settings, and the ability of these microorganisms to persist on hard surfaces, the role of contaminated surfaces in the transmission of these pathogenic species has frequently been under-discussed. In a clinical setting, where healthcare professionals are working alongside vulnerable patients, the elimination of virulent microorganisms is essential; despite this, many such microorganisms can be resistant to enhanced, deep-cleaning regimes^14^. Furthermore, another major issue is the development of antimicrobial resistance in the clinical environment. Levels of antimicrobial resistance have increased in recent years, and the potential for resistant strains to colonise hard surfaces, evade deep cleaning regimes, and continue to infect the most vulnerable patients is of ever-increasing concern^15^. In clinical settings, resistance is not just confined to antimicrobial agents–resistance has begun to emerge against biocidal agents, such as chlorhexidine and triclosan. Resistance to these biocides is frequently correlated with resistance to other antibiotics; for example, extensively-drug resistant *K. pneumoniae* and multidrug-resistant *Enterobacter* have been found to exhibit resistance to chlorhexidine^16,17^. As such, the development of novel antimicrobial technologies is vitally important, to prevent the colonisation of abiotic surfaces by pathogenic microorganisms, including those that are resistant to antimicrobial therapies, and to prevent patient infections by eliminating this mode of transmission.

One of the major issues around surface coatings as a method to prevent colonisation of abiotic surfaces, regardless of the materials used to produce them, is that they require synthesis in a factory or laboratory setting, often requiring specialised equipment and reagents^18^. This increases the costs of producing the materials, which in turn increases the costs of purchasing these antimicrobial technologies^19^. Due to the high costs, this restricts their widespread implementation, with the materials being reserved for specialist applications. As such, there is a need to develop alternative antimicrobial materials that can be applied to a pre-existing surface by the consumer. These types of materials will have lower production costs, which will make the purchasing costs lower; this will lead to more widespread applications of the material, including those that will benefit the general public.

With the need for intrinsically antimicrobial surfaces well documented, research has been carried out into the development of antimicrobial paints. Resins and paints containing silver nanoparticles appear to have potent antimicrobial efficacy against *E. coli* and *S. aureus*, although this activity was not quantified^20^. Other approaches have utilised copper nanoparticles to develop appliable antimicrobial materials, however the reduction in microbial burden was inconsistent across microbial species and application environment^21^. Recently, researchers have attempted to develop antimicrobial paints containing the plant-based compounds colophony and curcumin, which were found to provide a two-log reduction in the concentration of *S. aureus*, and a one-log reduction of *E. coli*, that could be recovered from painted surfaces; these values were not found to be statistically significant^22^. Other approaches have been based on cationic polymers, which were found to possess variable antimicrobial activity, with large quantities of highly concentrated polymeric paint being required to eliminate *E. coli* from surfaces. It was also found that the polymeric paints struggled to eliminate drug-resistant bacteria, including *K. pneumoniae*^23^. The ability to suspend the antiseptic agents chloroxylenol and terpineol in paint has also been investigated, with it being found that the antimicrobial activity of these paints is reduced when painted surfaces are exposed to deep cleaning techniques^24^. Despite these positive developments, many of these materials are still expensive to manufacture and purchase, with limited antimicrobial efficacy, and questions surrounding the durability of this efficacy. As such, it is crucial that antimicrobial paints continue to be developed, due to their greater affordability, but it is important that these paints possess significant, reproducible, and durable antimicrobial efficacy against a broad range of pathogenic microorganisms, including drug-resistant strains.

In this study, we demonstrate the development and characterisation of an industrial epoxy resin containing chlorhexidine digluconate (CHX). CHX is a broad-spectrum biocidal agent that exerts its effects by destabilising microbial membranes through the generation of reactive oxygen species, causing oxidative stress, lipid peroxidation, and leakage of metabolites from the cytoplasm, resulting in cell death^25–27^. Previous work has demonstrated the possibility of coating steel^28^, air filters^29^, and polymers^30^, with CHX, resulting in antimicrobial surfaces that possess strong antimicrobial efficacy, without altering the physical characteristics of the material. Previously, the coatings were all successfully characterised using time of flight secondary ion mass spectrometry (ToF–SIMS), specifically using the C_7_H_4_N_2_Cl^−^ (m/z 151.03) diagnostic secondary ion^31^. In this study, we aim to investigate the antimicrobial activity of CHX epoxy resin against clinically relevant pathogens *Staphylococcus aureus*, *Escherichia coli*, *Pseudomonas aeruginosa*, and *Candida albicans*. The effects of the addition of CHX to the physical properties of the paint, such as optical transparency and paint strength, will be investigated. We will also determine the durability of the antimicrobial efficacy of the paint and tested the efficacy of the paint against repeated bacterial exposures. Lastly, we will also examine if the paint remains biocidal against bacterial strains that have evolved resistance to CHX. This study represents an exciting development in the field of antimicrobial technologies, that could provide highly efficacious, durable, but low-cost, antimicrobial efficacy to pre-existing surfaces and materials.

## Materials and methods

All paint was provided by Indestructible Paint Ltd. All reagents were purchased from Sigma Aldrich UK, unless stated otherwise.

### Epoxy resin preparation

To prepare epoxy clear gloss resin, clear gloss base and epoxy catalyst were mixed in an equal ratio, and the chemicals were allowed to react together for 15 min. The resin was then applied in a thin layer (50 μm) to 1 cm × 1 cm steel samples, and the resin was allowed to cure until hard in a 60˚C incubator for 90 min.

To prepare chlorhexidine epoxy resin, CHX solution was added at a 1 in 10 dilution, to make a 2% CHX concentration (v/v). The reaction was allowed to proceed for 15 min as described above, and the resin was applied to steel samples for antimicrobial testing and aluminium samples for analytical testing and cured as previously described (60 °C, 90 min).

### Time-of-flight secondary ion mass spectrometry (ToF–SIMS)

All ToF–SIMS spectra of epoxy resin deposited on aluminium substrate were acquired using a ToF V (IONTOF GmbH) instrument. A 30 keV Bi_3_^+^ primary beam was used to acquire liquid metal ion gun (LMIG) ToF–SIMS images. The LMIG current was 0.05 pA, and the ToF image was run on an area of 300 µm × 300 µm using random raster mode with a 256 × 256-pixel density. The cycle time was set to 200 µs. The optimal target potential was set to −57.5 V. Four separate surface areas were analysed on each sample, and each measurement lasted 15 scans; the total ion dose per measurement was 9.44 × 10^10^ ions/cm^2^.

Depth profile measurement on the control resin sample and on chlorhexidine resin sample was carried out to determine chlorhexidine presence throughout the paint until the substrate is reached and the total ion dose was 3.59 × 10^16^ ions/cm^2^, which is approximated to be a 90 µm depth based on sputtering of irganox 1010 in similar conditions, which is commonly used as an analogous material to estimate the sputter yield of organic samples^32^. The depth profile analysis was carried out by using a 20 keV Ar_2072_^+^ gas cluster ion beam (GCIB) with a current of 4 nA as a sputter beam. An area of 300 µm × 300 µm was imaged during depth profiling, and the crater size was set to 500 µm × 500 µm. ToF–SIMS was also performed on post-leaching steel samples, with preparation of these samples described in the section below. The depth profile of the post-leaching samples was run until a total ion dose of 3.26 × 10^13^ ions/cm^2^.

Detection of the C_7_H_4_N_2_Cl^-^ ion using ToF–SIMS is used to confirm the presence of CHX on the surface, as this ion is a diagnostic fragment of the molecule of interest, CHX, as established in Judd, et al.^31^.

### Water contact angle

Epoxy resin deposited on aluminium substrate was prepared as described previously, and applied to steel surfaces and cured (60 °C, 90 min). The water contact angle was measured using the CAM 200 Goniometer (Krüss Scientific) using the sessile drop technique. 10 μL droplets of distilled, deionised water were used for the measurements, which were taken from 20 locations (five locations on each sample). The values for the water contact angle were obtained through application of Young–Laplace fitting to raw data.

### Scratch resistance and adhesion

To test the scratch resistance and adhesion strength of the paint, aluminium panels were shot blasted, before control and chlorhexidine epoxy resin was prepared as described above. Aluminium panels were painted with either type of epoxy resin using a 50 μm spiral wire bar coater (TQC Sheen) to obtain a thin, uniform paint layer. The painted sheets were then force cured at 121˚C for 60 min.

For scratch resistance, painted aluminium panels were placed in a manual scratch testing machine (TQC Sheen), and followed the assay according to ISO 1518^33^. In brief, weights were added to the apparatus, before being run across the painted surface until a scratch developed; the weight was increased in 100 g increments. The point at which to end the test was determined through examination with a magnifying glass, when it was observed that the integrity of the painted layer had been compromised.

For adhesion testing, a cross-cut test was performed. A 10 × 10 square pattern was scored into the painted aluminium steel using a template and a utility knife. A layer of tape was applied over the scored area, and was pulled to remove any paint that had not bonded to the aluminium sheet. The integrity of the paint was graded following the test according to ISO 2409:2007^33^.

### Optical transparency

Following preparation of control and chlorhexidine epoxy resin as described above, a thin layer (50 μm) of each type of resin was applied to the individual wells of a 96-well culture plate (Greiner Bio-One). The resin was cured as described previously. Once the resin was fully cured, 100 μL of phosphate buffered saline (PBS) was added to each well, and an absorbance spectrum reading taken using the BMG LabTech ClarioStar plate reader, scanning from 220 to 1000 nm.

### Antibacterial efficacy

*Escherichia coli* ATCC25922, *Staphylococcus aureus* ATCC6538 and *Pseudomonas aeruginosa* PA14 were cultured in 5 mL lysogeny broth (LB) for 18 h at 37˚C, 180 rpm, before being adjusted to 1 × 10^9^ CFU/mL. Steel samples that were painted with either control or antimicrobial epoxy resin were placed into a 12-well culture plate (Starlab). The cultures were pipetted onto the painted surfaces in 9 × 1 μL drops, in a simulated splash test. The cultures were allowed to dry onto the painted surfaces and were incubated on the surfaces for 18 h at room temperature, following the ISO standard 22196 ^33^. Following incubation, the surfaces were transferred into 10 mL Dey-Engley Neutralising Broth with 7–10 sterile zirconium beads (Thistle Scientific), and were vortex mixed for 1 min each to recover the bacteria from the surfaces. The neutralising broth suspension was serially diluted 1:3 in sterile PBS, and bacterial survival was determined by counting colony forming units (CFU) following incubation at 37 ˚C for 18 h on LB agar. Efficacy testing was performed in triplicate both for surfaces painted with control and antimicrobial epoxy resin, and for both *E. coli* and *S. aureus*.

### Antifungal efficacy

*Candida albicans* SC5314 was cultured in 5 mL yeast peptone dextrose (YPD) broth (1% yeast extract, 2% peptone, 2% glucose) for 18 h at 30˚C with shaking set to 180 rpm, before being adjusted to 1 × 10^9^ CFU/mL. Antimicrobial efficacy was then tested as described previously in **2.6**, replacing LB agar with YPD agar.

### Antibacterial efficacy against chlorhexidine-resistant *E. coli*

As detailed in Watson et. al. (2023), *E. coli* BW25113 was serially passaged in Mueller–Hinton Broth (MHB) containing increasing concentrations of CHX to elevate the minimum inhibitory concentration (MIC) of the strain to 32 μM ^30^. Chlorhexidine-resistant *E. coli* BW25113 was cultured overnight in 5 mL LB broth for 18 h at 37˚C, 180 rpm, before being adjusted to 1 × 10^9^ CFU/mL. Antibacterial efficacy was then tested as described previously in **2.6**.

### Scanning electron microscopy

Steel surfaces painted with control or chlorhexidine epoxy resin were prepared as described previously. *E. coli* and *S. aureus* were cultured in 5 mL LB for 18 h at 37˚C, 180 rpm, and then adjusted to 1 × 10^9^ CFU/mL. The samples were then placed into a 12-well culture plate (Starlab), with the cultures being pipetted onto the painted surfaces in 9 × 1 μL drops. The cultures were allowed to dry onto the painted surfaces and were incubated on the surfaces for 18 h at room temperature. Following this incubation, the surfaces were covered in 4% paraformaldehyde (Fisher Scientific) for one hour, before being washed with sterile PBS. The inoculated surfaces were then serially dried using solutions containing increasing concentrations of ethanol, up to 100% ethanol, and then underwent critical point drying. Samples were mounted using conductive carbon tape onto SEM support stubs and coated with Iridium (Quorum G 150 T ES, East Sussex UK). Scanning electron microscopy was performed at an operating voltage of 5 kV, nominal working distance 5 mm, SESI detector, using a field emission SEM (Zeiss CrossBeam 550, Oberkochen, Germany).

### Durability of antimicrobial efficacy

The durability of the antimicrobial efficacy of the paint was investigated by conducting a leaching study. Painted steel samples were prepared and cured as described previously. Following curing, the samples were immersed in 10 mL sterile PBS. All samples were stored in the dark, at room temperature. At 24-h intervals, 200 μL of the PBS solution was removed, and was plated in a black-bottomed, black-walled 96-well cell culture plate (Corning). After 14 days, the absorbance was read at 280 nm. The painted surfaces were removed from the leachate, and were dried and retained for future work. The leachate was also retained for future work, remaining in dark conditions.

To assess if there was any leaching of CHX from the painted surface, the antimicrobial efficacy of the leachate was assessed. The leachate was serially diluted 1:10 in sterile PBS, to a 10^–9^ concentration. 9 μL of *E. coli* at 1 × 10^9^ CFU/mL was added to 100 μL of each leachate dilution, and incubated at room temperature for one hour; fresh sterile PBS was used as a control, and was also inoculated with the bacterial suspension. The inoculated leachate solution was serially diluted 1:3 in sterile PBS, and bacterial survival was determined by counting CFU following incubation at 37˚C for 18 h on LB agar. Furthermore, to determine if the painted surfaces retained antimicrobial efficacy, *E. coli* was added to the surface as per **2.6**. The antimicrobial efficacy testing for both the leachate and the post-leaching painted surfaces was performed in triplicate.

### HPLC analysis of CHX epoxy resin leachate

Leachates from **2.10** were 0.22 μL filter sterilised into 2 mL amber robotic 9 mm thread vials for HPLC analysis. Chromatographic separation was achieved using an Agilent 1260 liquid chromatography system, consisting of a G7111B quaternary pump, G7115A diode array detector, G7129A vial sampler and the OpenLab, Agilent Technologies software version 3.5 (3.5.0.654). An ACE C18 (150 × 4.6 mm I.D.,5 μM) column was deployed using an isocratic mobile phase consisting of 32:68 (v/v) acetonitrile and phosphate buffer solution (0.05 M monobasic sodium phosphate solution including 0.5% triethylamine, which was adjusted to pH 3.0 using 85% phosphoric acid). A flow rate of 2.0 mL min-1 was consistent throughout the analysis, along with UV detection of 239 nm, a column oven temperature of 40 °C and an injection volume of 20 μL. A series of CHX standards varying from 0.1–1.0 mg mL^-1^ were used to develop a calibration curve for quantification of the CHX peak at retention 2.3 min.

### Stability of chlorhexidine epoxy resin

To determine the stability of chlorhexidine epoxy resin in shelf storage conditions, 20% (w/v) CHX solution was added to the clear gloss base to a concentration of 20% in a 15 mL centrifuge tube (Eppendorf), and mixed thoroughly. The tube was closed and wrapped in aluminium foil, to mimic shelf storage conditions, and the chlorhexidine epoxy base was stored at room temperature for one month. After this, the chlorhexidine epoxy base was mixed in an equal ratio with the epoxy catalyst, and the chemicals were allowed to react together for 15 min. The paint was applied to steel surfaces, and cured at 60˚C for 90 min. After this, the painted surfaces were investigated for antimicrobial efficacy. *E. coli* was cultured in 5 mL LB for 18 h at 37˚C, 180 rpm. The culture was adjusted to 1 × 10^9^ CFU/mL, and was applied to the painted surfaces in a 9 × 1 μL simulated splash pattern. The culture was allowed to dry onto the painted surfaces, and the surfaces were incubated overnight for 18 h at room temperature. Following this incubation period, antibacterial testing was performed as per 2.6, and bacterial survival was assessed by calculating CFU following incubation at 37˚C for 18 h on LB agar. Antibacterial testing for aged chlorhexidine resin was performed in triplicate.

This protocol was also performed for chlorhexidine epoxy base that had been stored at room temperature in the shelf-storage conditions described above for three months.

### Repeat exposures of chlorhexidine epoxy resin

Steel surfaces were painted with control or chlorhexidine epoxy resin, and cured as described previously. After this, the painted surfaces were investigated for the ability to retain antimicrobial efficacy upon repeat exposures to microorganisms. As such, *E. coli* was cultured in 5 mL LB for 18 h at 37˚C, 180 rpm. The culture was adjusted to 1 × 10^9^ CFU/mL, and was applied to the painted surfaces in a 9 × 1 μL simulated splash pattern. The culture was allowed to dry onto the painted surfaces, and the surfaces were incubated overnight for 18 h at room temperature. Following this incubation period, the painted steel samples were transferred to 10 mL sterile distilled water with 7–10 sterile zirconium beads (Thistle Scientific), and vortex mixed for 1 min to recover the bacteria from the surface. The bacterial suspension was serially diluted 1:3 in sterile PBS, and bacterial survival was assessed by calculating CFU following incubation for 18 h on LB agar. The painted steel pieces were removed from the bacterial suspension and washed in sterile distilled water, before being dried using an airline. After drying, the washed steel was inoculated again with a normalised culture of *E. coli*, and the bacterial recovery and washing stages were performed again, to a total of three exposures. Antibacterial testing was performed in triplicate for all three exposures.

### Data analysis

All statistical analysis was performed using SPSS (Version 28.0.1.1 (14); IBM). All data obtained was analysed by performing an Independent Samples t-test, with α set to 0.01. Image analysis was performed using ImageJ (Version 1.53t; NIH). All graphs were generated in Prism (Version 10.0.0 (131); GraphPad). All data is presented as the mean plus/minus the standard error of the mean.

## Results

### Surface characterisation

**Fig. 1:**
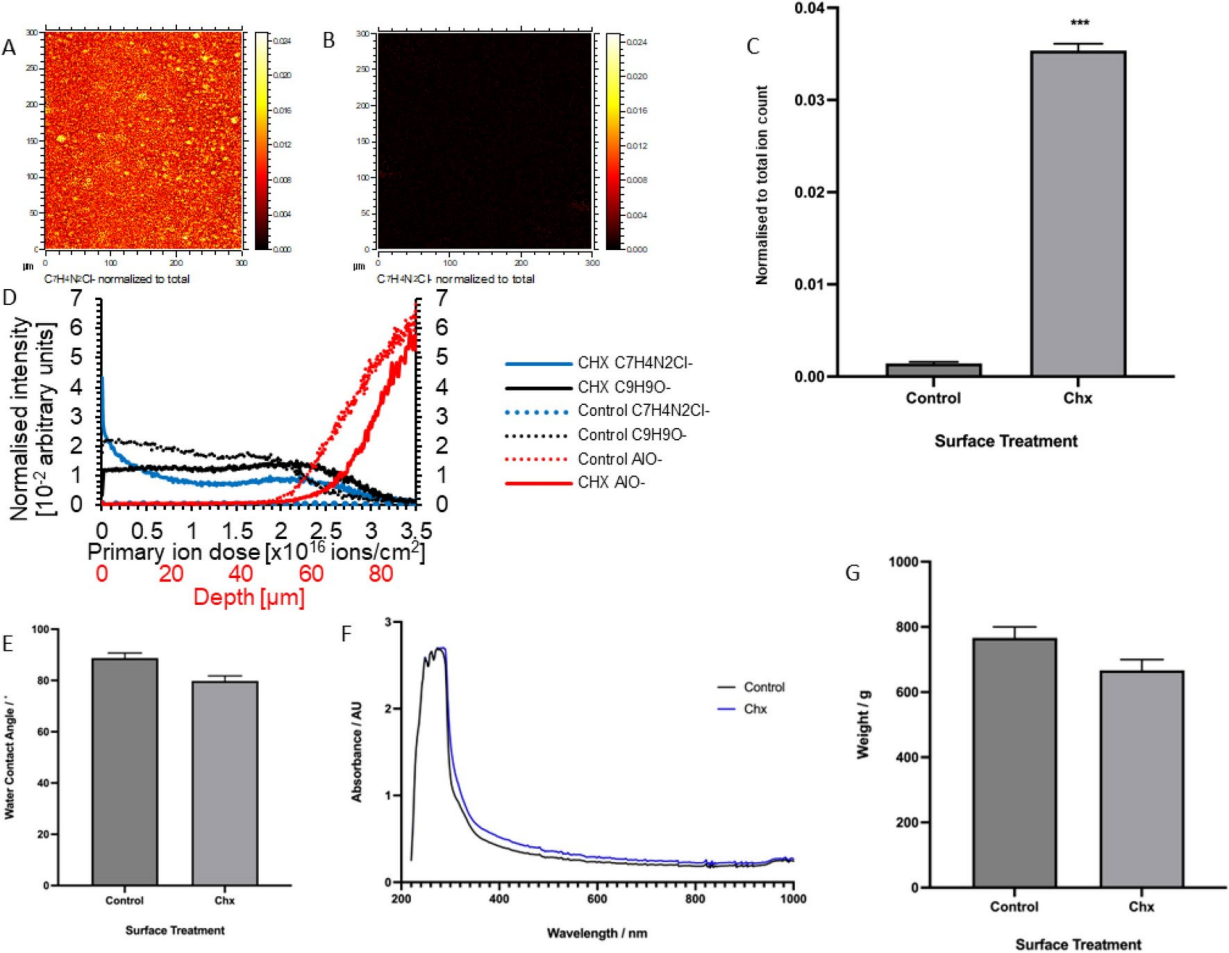
Surface characterisation of painted surfaces. (**A**) Scan across a steel surface painted with chlorhexidine epoxy resin, showing the intensity of the C_7_H_4_N_2_Cl^-^ ion normalized to total ion count. (**B**) Scan across a steel surface painted with control epoxy resin, showing the intensity of the C_7_H_4_N_2_Cl^-^ ion normalized to total ion count. (**C**) Quantification of C_7_H_4_N_2_Cl^-^ ion intensity across steel surfaces painted with control and chlorhexidine epoxy resin, respectively (n = 8). (**D**) The normalized ion intensity of the C_7_H_4_N_2_Cl^-^ ion (blue), C_9_H_9_O^-^ paint marker (black) and AlO^-^ substrate marker (red) through control (dotted line) and chlorhexidine epoxy resin (solid line). (**E**) Quantification of water contact angle measurements for steel surfaces painted with control and chlorhexidine epoxy resin and cured horizontally (n = 5). (**F**) The optical transparency of control and chlorhexidine epoxy resin, as evaluated by measurements of the absorbance from 220-1000 nm (n = 144). (**G**) The scratch resistance of control and CHX epoxy resin (n = 3). ***indicates *p* < 0.001, error bars show standard error of the mean.

ToF–SIMS was performed to detect the presence of CHX on the surface and throughout the epoxy resin (Fig. 1). Scans of the surfaces revealed uniformity of the presence of CHX ions across the surfaces painted with chlorhexidine epoxy resin (Fig. 1A), whilst these ions could not be detected on surfaces painted with control epoxy resin (Fig. 1B). The average ion intensity for surfaces painted with chlorhexidine epoxy resin was 0.035 ± 0.00073, which is significantly higher (*p* < 0.001) than the average ion intensity recorded for surfaces painted with control epoxy resin, which had a mean of 0.001 ± 0.00016 (Fig. 1C). In addition, ToF–SIMS depth profile was carried out on the control resin sample and on chlorhexidine resin sample to determine chlorhexidine (C_7_H_4_N_2_Cl^-^ ion) presence throughout the paint. Depth profiling through samples painted with chlorhexidine epoxy resin revealed that CHX ion was most prevalent at the uppermost layer, although it could be detected throughout the depth profile until the substrate was reached after delivering primary ion dose of approximately 2.7 × 10^16^ ions/cm^2^ (70 µm depth based on sputtering of irganox 1010 in similar conditions)^32^. (Fig. 1D). Lastly, the surface water contact angle was measured to determine if the addition of CHX to the epoxy resin altered the hydrophobicity of the material. Following curing, the addition of CHX to the epoxy resin decreased the water contact angle from 88.74 ± 1.97˚ to 79.83 ± 2.01˚. However, this decrease in the water contact angle was not found to be statistically significant (Fig. 1E), indicating that CHX can be incorporated into epoxy resin without significantly altering the wettability of the material.

### Material characterisation

The optical transparency of control and chlorhexidine epoxy resin was investigated alongside the curing time, finding that both the control and the CHX epoxy resin took 80 min to cure. After curing, the absorbance peaked at 274 nm for both control and chlorhexidine epoxy resin (Fig. 1F). This shows that the absorbance peak of epoxy resin is not altered by the presence of CHX. Despite the absorbance spectra following a similar pattern for all samples—absorbance peaking at 274 nm, and gradually decreasing as the wavelength increases—the absorbance of chlorhexidine epoxy resin was found to be increased at higher wavelengths in comparison to control epoxy resin. For example, at 1000 nm, the absorbance for control epoxy resin was 0.241 ± 0.0111 a.u., and was 0.266 ± 0.016 a.u. for chlorhexidine epoxy resin. This increase in absorbance at 1000 nm was not found to be statistically significant (n = 3, *p* = 0.14), indicating that CHX does not significantly increase the optical transparency of epoxy resin at higher wavelengths.

The scratch resistance of the two types of paint was determined. It was found that control epoxy resin was scratch resistant up to a weight of 766.67 ± 33.33 g, whereas chlorhexidine epoxy resin was scratch resistant up to a weight of 666.67 ± 33.33 g (Fig. 1G). Despite this observed decrease, this was not found to be statistically significant, and as such, it can be concluded that the addition of chlorhexidine to the epoxy resin does not significantly reduce the scratch resistance of the epoxy resin. As well as this, it was also determined that the addition of CHX did not impact the adhesion of the epoxy resin.

### Antimicrobial efficacy

**Fig. 2:**
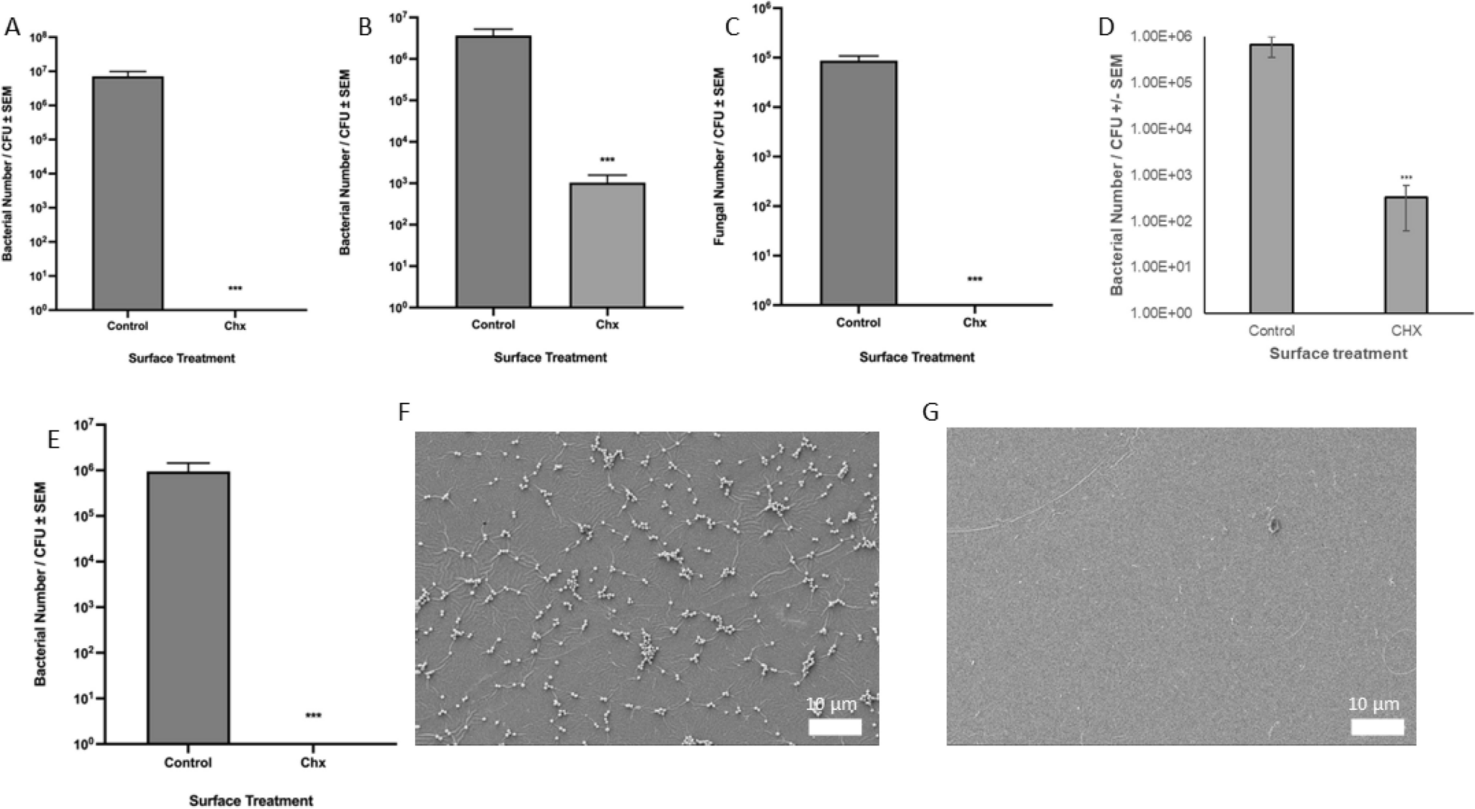
Antimicrobial efficacy of surfaces painted with chlorhexidine epoxy resin. (**A**) The survival of *E. coli* ATCC25922 on steel surfaces painted with control and chlorhexidine epoxy resin. (**B**) The survival of *S. aureus* ATCC6538 on steel surfaces painted with control and chlorhexidine epoxy resin. (**C**) The survival of *C. albicans* SC5314 on steel surfaces painted with control and chlorhexidine epoxy resin (**D**) The survival of *P. aeruginosa* PA14 on steel surfaces painted with CHX epoxy resin. (**E**) The survival of chlorhexidine-resistant *E. coli* BW25113 on steel surfaces painted with control and chlorhexidine epoxy resin (n = 9). ***indicates *p* < 0.001, error bars represent standard error of the mean. (**F**) SEM image of steel surface painted with control epoxy resin and inoculated with *S. aureus* ATCC6538. Image taken at 10 μm resolution and is representative of three repeats. (**G**) SEM image of steel surface painted with chlorhexidine epoxy resin and inoculated with *S. aureus* ATCC6538. Image taken at 10 μm resolution and is representative of three repeats.

With the ability to incorporate CHX into clear gloss epoxy resin established, and the presence of CHX on painted surfaces confirmed, the antimicrobial efficacy of the epoxy resin was next assessed. To replicate the contamination of abiotic surfaces observed in real-world scenarios, low-volume, high-concentration cultures (OD_600_ = 1.0) of *E. coli*, *S. aureus*, *P. aeruginosa* and *C. albicans* were used to inoculate the surfaces in a simulated splash pattern, as model Gram-negative, Gram-positive, and fungal organisms. For *E. coli*, following an overnight incubation, a CFU of 7.20 × 10^6^ ± 1.75 × 10^6^ CFU/mL was recovered from surfaces painted with control epoxy resin. By contrast, no viable bacteria could be recovered from surfaces painted with chlorhexidine epoxy resin. This was found to be highly statistically significant (*p* < 0.001) and is indicative of excellent antimicrobial efficacy (Fig. 2A).

Similar trends were observed for *S. aureus*; surfaces painted with control epoxy resin saw a CFU of 3.66 × 10^6^ ± 1.29 × 10^6^ CFU/mL recovered following an overnight incubation, whilst a CFU of 1.04 × 10^3^ ± 4.21 × 10^2^ CFU/mL was recovered from surfaces painted with chlorhexidine epoxy resin (Fig. 2B). This three-log reduction in CFU was found to be statistically significant (*p* < 0.001), and shows that the material exerts antibacterial efficacy against both Gram-positive and Gram-negative bacteria. The final bacterial species assayed was *P. aeruginosa*, and similarly, surfaces painted with control epoxy resin saw a CFU of 6.75 × 10^5^ ± 3.20 × 10^5^ CFU/mL recovered following an overnight incubation, whilst a CFU of 3.33 × 10^2^ ± 2.72 × 10^2^ CFU/mL was recovered from surfaces painted with CHX epoxy resin (Fig. 2D). This three-log reduction in CFU was found to be statistically significant (*p* < 0.001). The antifungal efficacy of the chlorhexidine epoxy resin was assessed utilising *C. albicans*. Following inoculation and incubation as described above, a CFU of 9.21 × 10^4^ ± 1.44 × 10^4^ CFU/mL was recovered from surfaces painted with control epoxy resin. By contrast, no viable fungi could be recovered from surfaces painted with chlorhexidine epoxy resin. This was found to be statistically significant (*p* < 0.001), indicating that the addition of chlorhexidine to epoxy resin confers antifungal efficacy (Fig. 2C).

Finally, to determine if the chlorhexidine epoxy resin could continue to exert its well-established antimicrobial efficacy in the event of the development of resistance to CHX, the surfaces were challenged with *E. coli* that had been evolved to possess a significantly elevated MIC against chlorhexidine, of 32 μM^30^. Following inoculation and incubation as described above, a CFU of 9.46 × 10^5^ ± 5.95 × 10^4^ CFU/mL was recovered from surfaces painted with control epoxy resin. By contrast, no viable bacteria could be recovered from surfaces painted with chlorhexidine epoxy resin. This was found to be highly statistically significant (*p* < 0.001), demonstrating that chlorhexidine epoxy resin retains its antimicrobial efficacy, even against strains that have evolved significant resistance to this biocidal agent (Fig. 2E).

This was also reflected in SEM images obtained from painted surfaces inoculated with *S. aureus* – a large concentration of bacteria was observed on surfaces painted with control epoxy resin (Fig. 2F), whilst little to no bacteria could be observed on surfaces painted with chlorhexidine epoxy resin (Fig. 2G).

### Surface leaching and durability of efficacy

**Fig. 3:**
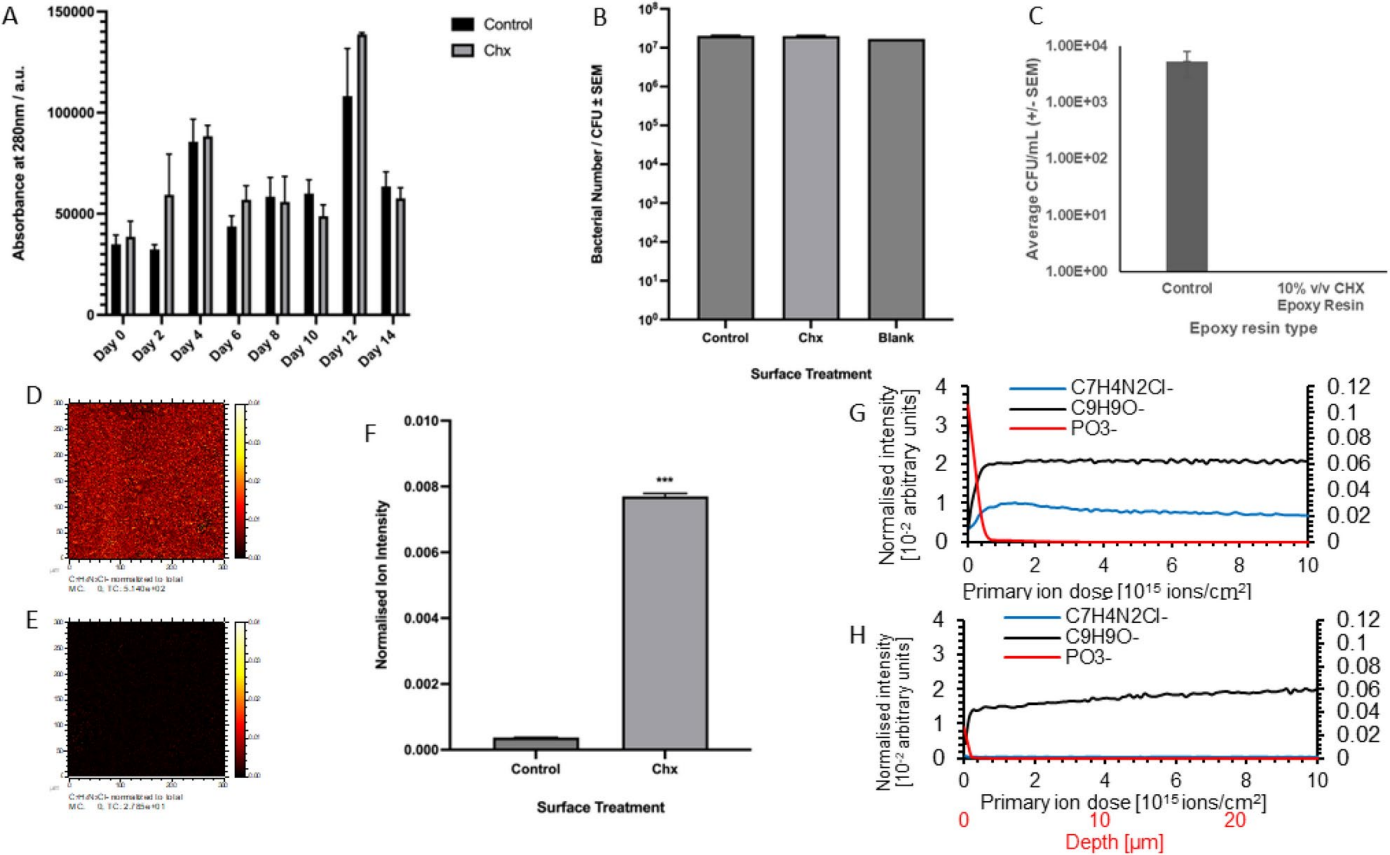
The effects of leaching in PBS on chlorhexidine epoxy resin. (**A**) The absorbance of leachate from steel surfaces painted with control and chlorhexidine epoxy resin (n = 9). (**B**) The antimicrobial efficacy of leachate obtained from steel samples painted with control and chlorhexidine epoxy resin (n = 3). (**C**) The survival of *E. coli* ATCC25922 on steel surfaces painted with control and chlorhexidine epoxy resin, following leaching (n = 9). (**D**) Scan across a steel surface painted with chlorhexidine epoxy resin following leaching, showing the intensity of the C_7_H_4_N_2_Cl^−^ ion. (**E**) Scan across a steel surface painted with control epoxy resin following leaching, showing the intensity of the C_7_H_4_N_2_Cl^−^ ion. (**F**) Quantification of C_7_H_4_N_2_Cl^−^ ion intensity across steel surfaces following leaching, painted with control and chlorhexidine epoxy resin, respectively (n = 8). (**G**) The normalized ion intensity of C_7_H_4_N_2_Cl^-^, C_9_H_9_O^−^, and PO_3_^−^ ions through successive layers of chlorhexidine epoxy resin following leaching in PBS. (**H**) The normalized ion intensity of C_7_H_4_N_2_Cl^−^, C_9_H_9_O^−^, and PO_3_^−^ ions through successive layers of control epoxy resin following leaching in PBS. In both chlorhexidine and control resins the PO_3_^−^ ion intensity is presented on secondary scale for clarity due to high intensity of the PO_3_^−^ peak. ***indicates *p* < 0.001, error bars show standard error of the mean.

Once the antimicrobial efficacy of the chlorhexidine epoxy resin was determined, the durability of this efficacy was investigated. The painted control and CHX surfaces were immersed in PBS for 14 days. On day 0, background readings were taken before adding painted samples: for control epoxy resin leachate, the average day 0 reading was 36,772 ± 3579 au, and was 38,676 ± 4428 au for chlorhexidine epoxy resin leachate. Throughout the study, the absorbance readings fluctuated, with increases and decreases being observed for leachate obtained from surfaces painted with both control and chlorhexidine epoxy resin, respectively. No significant differences were found between any of the control and chlorhexidine leachate absorbance readings for any of the measurements, indicating that there was no leaching of chlorhexidine from the epoxy resin upon immersion in PBS (Fig. 3A).

To confirm that there was no CHX leaching from the epoxy resin, the antimicrobial efficacy of the leachate was assessed. For the leachate from the control surfaces, a bacterial concentration of 2.04 × 10^7^ ± 4.02 × 10^6^ CFU/ml was recovered, whilst for the leachate from the chlorhexidine-painted surfaces, the CFU was 1.85 × 10^7^ ± 1.06 × 10^6^ CFU/ml. It was found that there was no significant difference between the two bacterial concentrations (*p* = 0.666) (Fig. 3B). The leachate from the chlorhexidine-painted surfaces does not exhibit any antimicrobial efficacy, which indicates that there is no leaching of the CHX from the surface. This was confirmed with HPLC analysis of the leachate, and compared to a CHX standard, and no CHX was detected in the leachate from either the CHX epoxy resin or control epoxy resin. It can therefore be concluded that the chlorhexidine remains suspended inside the epoxy resin, and indicates that the antimicrobial efficacy is durable.

Lastly, following the leaching assay, the painted surfaces were removed from the leachate, and the post-leaching surfaces were inoculated with *E. coli*. The results showed that a bacterial concentration of 5.33 × 10^3^ ± 2.62 × 10^3^ CFU/mL was recovered from control surfaces, whilst no viable *E. coli* were recovered from surfaces painted with chlorhexidine epoxy resin (Fig. 3C). This was found to be highly significant (p = 0.022), indicating that the painted surfaces remain antimicrobial following leaching, which is supported by the other data obtained in this study, demonstrating that CHX remains within the epoxy resin.

After the samples were removed from the leachate and dried, ToF–SIMS was performed, to see if chlorhexidine could continue to be detected in the epoxy resin following leaching. It was found that the C_7_H_4_N_2_Cl^-^ ion could be detected uniformly across a layer painted with chlorhexidine epoxy resin (Fig. 3D), whilst it could not be detected in samples painted with control epoxy resin (Fig. 3E). The average ion intensity for samples painted with chlorhexidine epoxy resin was found to be 0.0077 ± 0.0000044, whereas this ion intensity was 0.00038 ± 0.0000041 for samples with control epoxy resin; this difference was found to be highly statistically significant (*p* < 0.001) (Fig. 3F). This shows that CHX remains incorporated in epoxy resin after leaching. ToF–SIMS analysis also revealed that, following leaching, a high concentration of phosphate salt ions could be detected on surfaces painted with control and chlorhexidine epoxy resin, respectively (Fig. 3G,H). This confirms that the immersion of painted surfaces in PBS does not prevent the chlorhexidine epoxy resin from acting as an antimicrobial material.

### Stability of chlorhexidine in epoxy resin

**Fig. 4:**
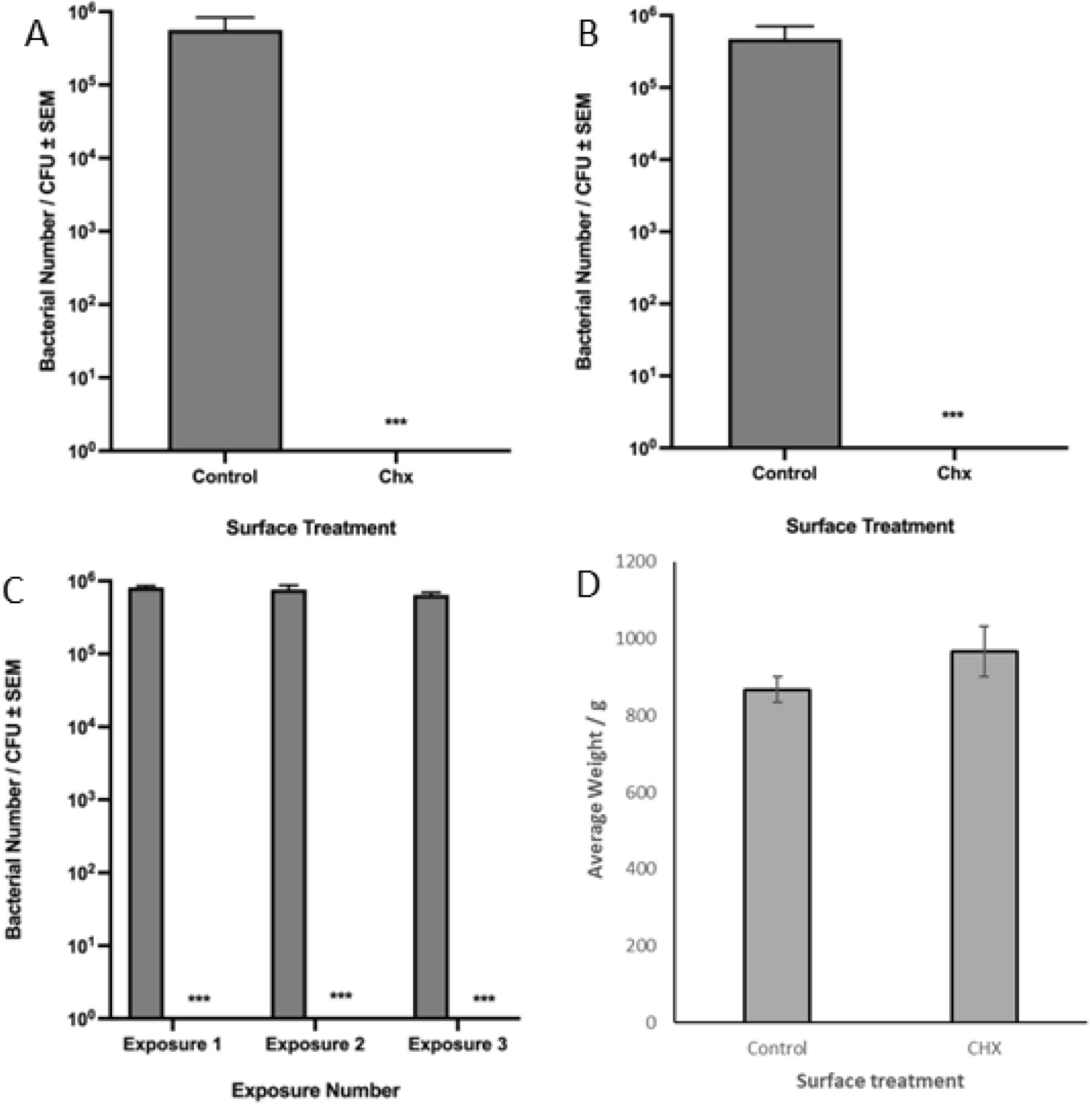
Continued explorations of the antimicrobial efficacy of chlorhexidine epoxy resin. (**A**) The survival of *E. coli* ATCC25922 on steel surfaces painted with control epoxy resin, or surfaces painted with one-month aged chlorhexidine epoxy resin (n = 9). (**B**) The survival of *E. coli* ATCC25922 on steel surfaces painted with control epoxy resin, or surfaces painted with three-month aged CHX epoxy resin (n = 9). (**C**) The survival of *E. coli* on steel surfaces painted with control and CHX epoxy resin without sterilization between repeat exposures (n = 9). (**D**) The scratch resistance of both control and CHX epoxy resin, after 14 days leaching in PBS (n = 3). ***indicates *p* < 0.001, error bars indicate the standard error of the mean.

To determine the ability to store chlorhexidine epoxy resin under shelf-storage conditions, we mixed CHX into the epoxy base, and kept the mixture in dark conditions for one month. After this, the chlorhexidine base was mixed with the catalyst, and applied to steel surfaces for the determination of antimicrobial efficacy. Following inoculation and incubation, a bacterial concentration of 5.64 × 10^5^ ± 9.50 × 10^4^ CFU/mL was recovered from surfaces painted with control epoxy resin. Conversely, no viable bacteria could be recovered from surfaces painted with the aged chlorhexidine epoxy resin; this was found to be highly statistically significant (*p* < 0.001) (Fig. 4A). This shows that the excellent antimicrobial efficacy of the paint is not affected by storing for extended periods of time, and that the material could be stored on the shelf without sacrificing antimicrobial efficacy. The experiment was repeated with chlorhexidine base that had been stored for three months, and it was found that a bacterial concentration of 4.72 × 10^5^ ± 3.77 × 10^4^ CFU/mL was recovered from surfaces painted with control epoxy resin. By contrast, no bacteria could be recovered from surfaces painted with chlorhexidine epoxy resin prepared with the three-month aged base (Fig. 4B). This was found to be highly statistically significant (*p* < 0.001), demonstrating that the material can be stored for three months without adversely affecting the well-demonstrated antimicrobial efficacy of the material. Furthermore, scratch testing the painted surface post-leaching also showed that aging the surface not only has no effect on the antimicrobial efficacy, but also no significant impact on the surface integrity itself (p = 0.197) (Fig. 4D).

### Repeated exposures of chlorhexidine epoxy resin to *E. coli*

To observe if the chlorhexidine epoxy resin was able to exert its antimicrobial efficacy in a realistic setting, the painted surfaces were challenged with multiple exposures to *E. coli* without sterilisation in between exposures. On the first exposure, a bacterial concentration of 8.13 × 10^5^ ± 4.31 × 10^4^ CFU/mL was recovered from surfaces painted with control epoxy resin, with no viable bacteria being recovered from surfaces painted with chlorhexidine epoxy resin. After the second exposure, a bacterial concentration of 7.62 × 10^5^ ± 1.10 × 10^5^ CFU/mL was recovered from surfaces painted with control epoxy resin, whilst, once again, no viable bacteria could be recovered from surfaces painted with chlorhexidine epoxy resin. After the third and final exposure, a bacterial concentration of 6.44 × 10^5^ ± 5.12 × 10^4^ CFU/mL was recovered from surfaces painted with control epoxy resin, whilst no bacteria could be recovered from surfaces painted with chlorhexidine epoxy resin. The bacterial concentration was significantly reduced for chlorhexidine epoxy resin in comparison to control epoxy resin after each exposure (*p* < 0.001 for each exposure), indicating that the antimicrobial efficacy of the chlorhexidine epoxy resin is not diminished by repeatedly exposing the material to *E. coli* (Fig. 4C).

## Discussion

In this study, we have shown the ability to impregnate commercially available epoxy resin with the broad-spectrum biocidal agent CHX. Detection of the C_7_H_4_N_2_Cl^−^ ion using ToF–SIMS is used to confirm the presence of chlorhexidine in a material^31^. The detection of this ion confirms the presence of chlorhexidine within the epoxy resin, and shows that the distribution of this ion is uniform across the painted layer, whilst the ion could not be detected in control samples. Further analysis revealed that, despite the ion being detected throughout the applied layer of epoxy resin, it predominantly accumulates at the surface. The reason for this is unclear, although it could potentially be due to phase separation. The preferential aggregation of chlorhexidine at the uppermost layer of the applied epoxy resin upon curing may enhance the antimicrobial efficacy of the material, as this would ensure constant contact between the pathogen and the biocide; this is important, as CHX exerts its antimicrobial effects through contact killing mechanisms and interactions with the microbial membrane^34,35^. This would lead to effective clearance of the microorganism from the surface, preventing colonisation and future transmission. Further investigations found that the addition of CHX to epoxy resin led to minimal alterations to the water contact angle, optical transparency, and bonding and adhesion strength of the epoxy resin. Demonstrating that the incorporation of CHX does not adversely affect the well-established properties of epoxy resin^36^, and that the material can be used by the consumer in the same manner as control epoxy resin.

The antimicrobial efficacy results show that CHX epoxy resin exhibits significant antimicrobial activity against both Gram-positive and Gram-negative bacteria, although the antimicrobial efficacy was stronger against *E. coli* (Gram-negative) than against *S. aureus* (Gram-positive), as determined through surface testing and SEM imaging of inoculated surfaces. Previous research has suggested that CHX is most effective against Gram-positive bacteria; as such, more research will need to be undertaken to understand why the converse is true for the CHX epoxy resin we have developed^37^. CHX epoxy resin was also highly efficacious against pathogenic *C. albicans*, indicating that the broad-spectrum biocidal activity of CHX is not diminished by suspension in epoxy resin. Lastly, the CHX epoxy resin was also highly effective against prolific biofilm former, *P. aeruginosa*. The exhibited antimicrobial efficacy was repeatable and significant, indicating that this technology could be used in real-world settings as a potent biocide. This indicates that the epoxy resin can be applied to a variety of pre-existing surfaces without affecting the biocidal action of the suspended CHX, due to the flexibility afforded by the ease of application and curing.

With the importance of antimicrobial resistance well established, and the knowledge that resistance to chlorhexidine has been observed in clinical settings, it is important to determine if the chlorhexidine epoxy resin we have developed will continue to exert its antimicrobial efficacy even if resistance arises. As such, we tested the material against a strain of *E. coli* that was engineered to have a significantly elevated MIC against chlorhexidine. The MIC of this evolved strain was 28.7 μg/mL; this is significantly greater than the MIC value of 0.9 μg/mL that wild-type *E. coli* possesses^38^. Furthermore, this is a greater MIC value than is considered the threshold for chlorhexidine resistance, which is typically cited as an MIC greater than 4 μg/mL^39^. Despite this, the chlorhexidine paint was still highly effective, with no viable bacteria being recovered from the paint surface after inoculation and incubation. This is a highly positive development, and suggests that the material could be used to provide low-cost antimicrobial efficacy to pre-existing surfaces, and will continue to do so when colonised by chlorhexidine-resistant strains. This ensures that multidrug-resistant strains of pathogenic microorganisms will be eliminated from surfaces painted with the chlorhexidine epoxy resin, preventing the transmission of these dangerous pathogenic species from abiotic surfaces to the most vulnerable.

In addition to this, it is of the utmost importance that the antimicrobial efficacy of the material is durable; this was assessed by determining the efficacy of the chlorhexidine epoxy resin after 14 days immersion in sodium chloride solution. This environment places the chlorhexidine epoxy resin under stress conditions, as the diffusion of water into the polymer leads to the irreversible chemical degradation of the epoxy resin in a process known as plasticisation. As such, it is not recommended to expose epoxy resin materials to water for extended periods of time^40,41^. It was found that the material continued to exert excellent antimicrobial efficacy against *E. coli*, demonstrating that the antimicrobial efficacy is durable, indicating that the chlorhexidine epoxy resin would remain biocidal for extended application times. Previous studies that have incorporated chlorhexidine into epoxy resin materials for antimicrobial coatings have relied on the principle of diffusion of chlorhexidine out of the epoxy resin in order to exert antimicrobial efficacy^42–44^. By contrast, we have developed a paintable material in which the CHX remains stably incorporated into the epoxy resin, without leaching of the biocide from the painted layer, as demonstrated by the retained antimicrobial efficacy and HPLC. This indicates that the material is suitable for long-term applications, without the need to reapply the painted layer.

We have shown that the chlorhexidine retains its antimicrobial efficacy when mixed into the epoxy resin base, and stored at room temperature for three months. This is of the utmost importance, as a product would be kept in shelf storage conditions when it is commercially available, until purchased by the consumer. As many antimicrobial and biocidal paints and coatings have been found to be unstable under extended storage conditions^45–48^ the evidence of the storage stability of chlorhexidine epoxy resin, and the retention of its well-established antimicrobial efficacy under these extended storage conditions, has excellent implications for the implementation of this material in real-world settings. As well, further suggests that the material could successfully be made available commercially for purchase by the consumer. We have also shown that the chlorhexidine epoxy resin is highly effective against repeat exposures to pathogenic microorganisms without sterilisation in between inoculations. This is important, as previous studies have shown that surfaces can continue to be contaminated by a variety of pathogenic microorganisms even after cleaning has taken place^49^. Furthermore, many abiotic surfaces do not undergo regular deep cleaning, leading to continual contamination of the surface environment^50^. As such, this shows that the paint will be effective in a real-world setting, being capable of eliminating pathogenic microorganisms from the surface environment when enhanced hygiene measures are not being implemented.

To conclude, we show the ability to suspend CHX in epoxy resin, and that the distribution of CHX across the exposed, painted layer of this material is uniform. The addition of CHX was found to have little effect on the water contact angle of painted surfaces, and to have a minimal effect on the optical transparency of the paint upon curing. The material also had bonding strength and scratch resistance comparable with control samples. The resulting epoxy resin was found to possess a significant, reproducible antimicrobial effect against Gram-positive and Gram-negative bacteria, and against pathogenic fungi. It was also found that the paint continues to exert its antimicrobial efficacy when challenged with bacteria evolved to exhibit resistance to chlorhexidine. Chlorhexidine epoxy resin was found to be highly effective against pathogenic microorganisms when challenged with repeat inoculations without sterilisation, and that the paint can be stored for extended periods without a loss of biocidal activity. It was also found that the antimicrobial efficacy of the paint was durable, and is suitable for long-term applications without sacrificing its biocidal activity. We believe that these developments could be used by companies and individuals to provide highly effective, but affordable, antimicrobial surfaces, to eliminate the role of the contamination of abiotic surfaces in the transmission of infection, with a broad range of applications. Following on from this work, we shall be investigating the possibility of aerosolising chlorhexidine epoxy resin, and shall also determine what concentration of CHX is required to confer antimicrobial efficacy to epoxy resin as a minimum. These steps will reduce the costs of synthesis, reducing costs to the consumer. We shall also be investigating the possibility of impregnating a water-based epoxy resin with CHX. Epoxy resin materials that do not contain an organic solvent base are more environmentally friendly, and altering the base material will be of significant commercial benefit.

## Data Availability

Data is stored in the University of Nottingham repository.
